# Rewiring of the Transcription Factor Network in Acute Myeloid Leukemia

**DOI:** 10.1177/1176935119859863

**Published:** 2019-06-25

**Authors:** Salam A Assi, Constanze Bonifer, Peter N Cockerill

**Affiliations:** Institute of Cancer and Genomic Sciences, University of Birmingham, Birmingham, UK

**Keywords:** Acute myeloid leukemia, transcription factor, network, gene regulation, gene expression, DNase, DNA mutation, FLT3, AP-1, RUNX1, CEBPA

## Abstract

Acute myeloid leukemia (AML) is a highly heterogeneous cancer associated with different patterns of gene expression determined by the nature of their DNA mutations. These mutations mostly act to deregulate gene expression by various mechanisms at the level of the nucleus. By performing genome-wide epigenetic profiling of cis-regulatory elements, we found that AML encompasses different mutation-specific subclasses associated with the rewiring of the gene regulatory networks that drive differentiation into different directions away from normal myeloid development. By integrating epigenetic profiles with gene expression and chromatin conformation data, we defined pathways within gene regulation networks that were differentially rewired within each mutation-specific subclass of AML. This analysis revealed 2 major classes of AML: one class defined by mutations in signaling molecules that activate AP-1 via the mitogen-activated protein (MAP) kinase pathway and a second class defined by mutations within genes encoding transcription factors such as RUNX1/CBFβ and C/EBPα. By identifying specific DNA motifs protected from DNase I digestion at cis-regulatory elements, we were able to infer candidate transcription factors bound to these motifs. These integrated analyses allowed the identification of AML subtype-specific core regulatory networks that are required for AML development and maintenance, which could now be targeted in personalized therapies.

**Comment on:** Assi SA, Imperato MR, Coleman DJL, et al. Subtype-specific regulatory network rewiring in acute myeloid leukemia. *Nat Genet*. 2019;51(1):151-162. doi:10.1038/s41588-018-0270-1. PubMed PMID:30420649. https://www.ncbi.nlm.nih.gov/pubmed/30420649

## Distal Regulatory Elements and DNA Motifs Can Be Used to Build Gene Regulation Networks

The regulation of transcription in development is coordinated by specific combinations of different transcription factors (TFs) that cooperate in the activation of gene promoters and their distal cis-regulatory elements. TFs coordinate many of the functions and fates of cells, from development to proliferation and differentiation, and in response to external signals. Any interference in TF-driven developmental programs can potentially trigger the onset of tumorigenesis. TFs are grouped into distinct classes based on their DNA-binding domains (DBDs),^1^ and whole families of TFs typically share essentially identical DNA-binding motifs. The activation of gene expression by distal regulatory elements involves cooperation between multiple TFs and chromatin modifiers that form large multi-protein complexes that replace nucleosomes, generating accessible open chromatin regions.^2^ Gene regulation networks (GRNs) involve the binding of TFs to each other’s genes as well as to downstream targets and can be inferred by identifying the motifs for TF families within specific subsets of open chromatin regions representing potential cis-regulatory elements.^3^ Such elements are typically defined as DNase I hypersensitive sites (DHSs) using either DNase I or transposase (ATAC) to probe accessible regions.^2,4^ The interactions between specific TFs and their target genes form specific regulatory nodes within the GRNs that define cell types and their stages of differentiation. Rapid advances in next-generation sequencing and computational modeling together with the rapidly increasing volume of genome-wide data deposited on public databases provide opportunities for high-quality construction of TF networks. Direct approaches, such as chromatin immunoprecipitation with sequencing (ChIP-Seq),^5^ enable the identification of sites bound by specific individual TF family members in living cells and provide valuable insights into gene regulation mechanisms.

## Subtype-Specific Gene Regulatory Networks in Acute Myeloid Leukemia

Acute myeloid leukemia (AML) is a cancer that develops in a step-wise manner via the accumulation of different classes of DNA mutations starting in blood stem cells that go on to form pre-leukemic cells that eventually progress to AML.^6^ The evolution of cancer cells typically follows the course of either mutations in epigenetic modifiers, such as *DNMT3A, TET2*, or *IDH1/2*, which can promote clonal expansion of stem cells,^7^ or mutations in genes controlling differentiation, such as *RUNX1* and *CEBPA*, followed by mutations in signaling molecules such as RAS family genes, and cytokine receptors that include *KIT* and *FLT3*, which activate RAS to promote proliferation and block apoptosis.^8^ Signaling mutations, such as *FLT3-Internal Tandem Duplication* (ITD), typically cause constitutive signaling activation, whereas TFs usually acquire loss or change of function mutations.

It was previously established that AML gene expression profiles cluster according to the mutation subclass,^9^ but the GRNs underlying these patterns were unknown. To crack this code, it was necessary to delve into the nucleus and determine what specific classes of TFs were associated with which DNA motifs within deregulated cis-regulatory elements. We initially pioneered this approach by focusing on AML associated with single classes of mutation. For both FLT3-ITD^10^ and t(8;21)^11^ AML, we identified common sets of distal elements and occupied TF motifs associated with specific patterns of aberrant gene expression. For FLT3-ITD AML, chronic mitogen-activated protein (MAP) kinase signaling led to the activation of genes bound by the AP-1 and RUNX1 TFs. In the case of t(8;21) AML, the RUNX1-ETO fusion protein acted as a repressor to block the expression of C/EBPα target genes and, thereby, differentiation to macrophage lineage cells.

Our recent AML study by Assi et al^12^ was more ambitious and used multiple types of analyses and data to identify TF networks in several different mutation subtypes of AML and then compared them with each other. Previous studies of gene regulation have designated many of the features of TF networks and developed technology to study them. Kang et al^13^ described a TF network mapping algorithm based on gene expression data named as NetProphet 2.0, which combined different expression-based network models for better results. This algorithm accounts for the fact that TFs with similar DBDs with similar amino acid sequences are likely to bind similar sets of target genes. It also identified motifs that are present in the promoters of target genes and distinguished likely target genes from unlikely target genes to improve the network map. Parra et al^14^ developed the web server “INSECT” to predict the occurrence of cis-regulatory modules which control gene expression using position weight matrices (PWMs) to search for TF motifs within a certain distance relative to the transcription stat sites (TSS) for groups of genes. Kulkarni et al^15^ also used the PWM approach to predict TF regulators and construct gene regulatory networks in *Arabidopsis*. Ramirez et al^16^ integrated the ATAC-Seq and RNA-Seq data to generate dynamic gene regulatory networks using time course data from the human HL60 cell line model of myeloid differentiation. Goode et al^17^ integrated data from RNA-Seq, DNaseI-Seq, and ChIP-Seq for histone marks and TFs to show that multiple dynamic changes to TF networks take place during the differentiation of embryonic stem cells into macrophages. A common feature of such studies is combining gene expression data with either TF motif searches using PWMs^13^ or using TF binding sites identified by ChIP-Seq experiments. One limiting feature of these studies is that they typically define TF networks using cell line models. More limited studies have been based on primary AML samples, mostly using gene expression data only, such as The Cancer Genome Atlas (TCGA) study^18^ and the Valk et al^9^ study.

Assi et al^12^ determined how the disruption of specific TF function and aberrant signaling leads to an altered specific pattern of aberrant chromatin programming and changes in gene expression in AML. To achieve this task, it was necessary to integrate multiple types of genome-wide data. We addressed this question by performing a comprehensive set of genome-wide analyses on transcriptome by RNA-Seq, digital footprinting by DNaseI-Seq, and chromatin conformation capture data. This study used a cohort of 32 AML samples obtained from highly purified populations of undifferentiated cells comprising at least 90% leukemic blasts from AML patients with distinct TF and signaling molecule mutations to define the mechanisms of AML-subtype-specific regulatory circuitries. To eliminate potentially confounding samples with a significant level of subclonal populations, we also ensured that the allelic frequency of each mutation was close to the 50% level expected for a clonal population, or 100% where both alleles were mutated. Acute myeloid leukemia samples were compared with non-malignant CD34^+^ mobilized peripheral blood stem cells (PBSCs) and to published data from progenitor cells at different stages of lineage commitment.^19^

**Figure 1.**
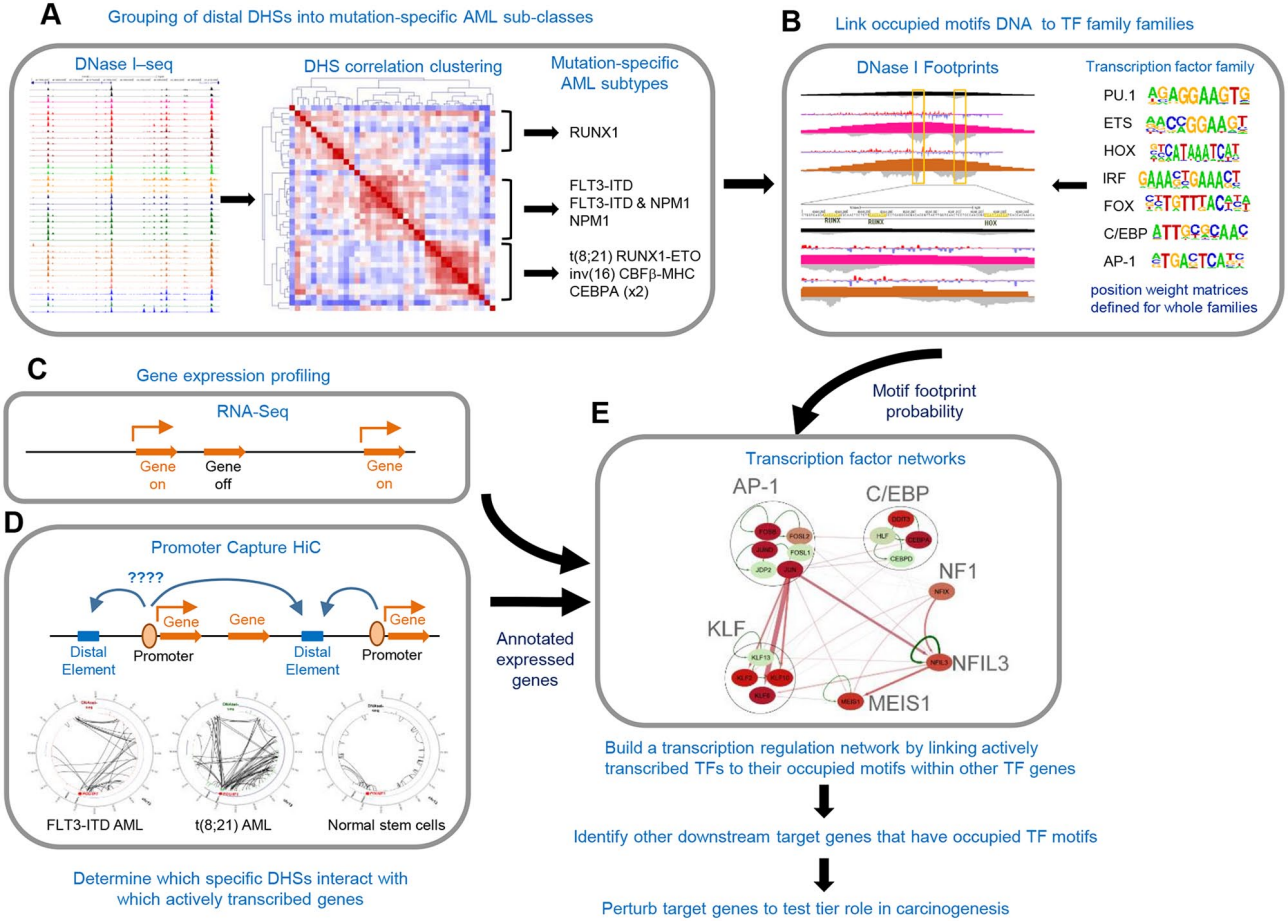
Overview of the AML mutation-specific TF network pipeline. Global mapping of DHSs allows for the identification of discrete subsets of DHSs associated with specific classes of mutations. High-read depth DNaseI-Seq can be used to infer occupancy of TF motifs within DHSs. Parallel RNA-Seq data can be used to identify which potential specific TF family members are likely to be bound to the occupied TF motifs. Regulatory networks can then be constructed using promoter capture HiC data to infer which DHSs and TFs are likely to be regulating which active genes. AML indicates acute myeloid leukemia; DHS, DNase I hypersensitive site; TF, transcription factor.

Figure 1 provides an overview of the AML-mutation-specific TF network pipeline used by Assi et al. In step A of Figure 1, high-depth DNaseI-Seq data (an average of more than 200 million sequence reads) were generated from all 32 AML samples, with several samples for each mutation-specific subset. An all-inclusive high-confidence set of ~128 000 distal DHS peaks was then defined by first merging the aligned reads from all samples prior to peak calling. This approach was designed to maximize the precision and sensitivity of peak detection and flatten the background noise, thereby greatly reducing the levels of both false-negative and false-positive DHS detection. Using this approach, we detected a median of ~32 000 distal DHSs per sample and normalized the data using the value for the peaks ranked by size at 16 000 which was close to the median in each case. Unsupervised hierarchical clustering was then performed using the same set of 400-bp windows for all 128 000 regions in each sample. This analysis revealed that just 7 classes of mutations allowed the patients to be divided into 3 major subsets, and additional minor subsets sharing very similar DNaseI-Seq profiles (step A in Figure 1). Surprisingly, these profiles were defined based on signaling and TF gene mutations alone, whereas commonly mutated epigenetic regulators, such as *DNMT3A, TET2*, and *IDH1/2*, had essentially no impact on driving specific patterns of gene expression. This was perhaps unsurprising given that, apparently, normal clonal hematopoiesis can arise driven by such mutations.^7^ By also comparing the AML-specific chromatin profiles with different types of progenitor cells,^19^ we demonstrated that these cells were not simply blocked at a specific stage of myeloid differentiation but were rewired in different directions altogether, acquiring different identities. The BLUEPRINT consortium, using a different patient cohort, recently reached similar conclusions and identified 2 major subsets defined by either (1) *NPM1* or *FLT3* mutations or (2) *RUNX1* or splicing factor mutations.^20^ Interestingly, splicing factor mutations were also prominent in the RUNX1 mutation cluster identified by Assi et al.

**Figure 2.**
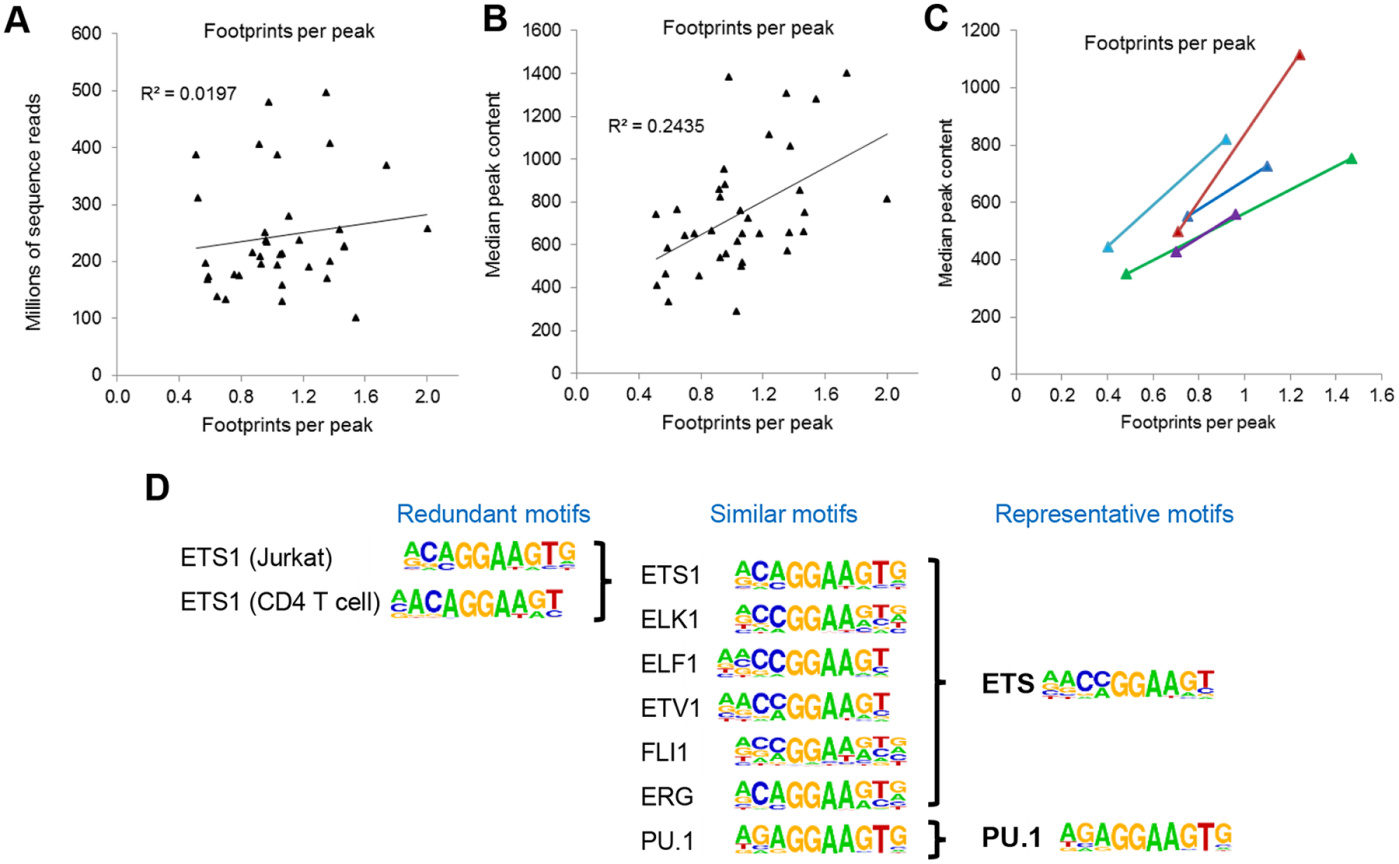
Optimization of data used to construct regulatory networks. (A-C) Efficient identification of occupied motifs using Wellington requires high-read depth DNaseI-Seq data. Due to the variability between DNaseI-Seq data sets, the read depth alone is insufficient to predict the depth of sequencing required to reliably predict footprints (A). In contrast, the median DHS peak volume is a more reliable guide, with a peak volume of ~700 being able on average to allow identification of 1 footprint per peak. Because Wellington is based on statistical probabilities, the likelihood of identifying footprints also increases in the proportion to overall sequence depth (C). (D) The linking of expressed TFs with their DNA motifs is critically dependent on knowing which motifs are likely to be bound by which TFs. Such analyses are often confounded by the overabundance of different motifs ascribed to the same factor, or ascribed to individual family members that bind to the same motifs. We circumvented this problem by identifying single representative motifs that can be associated with entire subgroups of TFs which bind the same motifs and splitting TF families into subsets based on any substantial differences in PWMs. (D) Illustration of this process using annotated HOMER^22^ and JASPAR motifs to identify similarities and differences among motifs for ETS family TFs. HOMER motifs are taken from http://homer.ucsd.edu/homer/motif/HomerMotifDB/homerResults.html and JASPAR motifs are taken from http://jaspar.genereg.net/. DHS indicates DNase I hypersensitive site; PWM, position weight matrix; TF, transcription factor.

In step B of Figure 1, the DNaseI-Seq data were further processed to identify the occupied TF motifs using the Wellington digital footprinting algorithm,^21^ as used in our previous FLT3-ITD and t(8;21) AML analyses,^10,11^ but not in most other AML studies. As an additional vital step in the quality control for DNaseI-Seq, we first determined what sequence depth was required to achieve consistent and reliable footprinting data. We initially noticed that sequence depth alone was a poor predictor of how much data were needed to efficiently detect footprints (Figure 2A), presumably due to variations in background noise. In contrast, the median number of reads present in the peaks was a much better indicator, with a peak content of ~700 reads being needed for the detection of an average of 1 footprint per peak (Figure 2B). For 5 AML samples where footprint detection was initially inefficient, we were able to increase the sequencing depth (to 190-280 million reads) to a point where we could detect in the order of 1 footprint per peak (Figure 2C).

As most TFs within the same family recognize essentially identical DNA sequences, we needed to compile a non-redundant database of representative motifs covering each TF family. Transcription factor network analyses can otherwise be confounded by the fact that different TF-specific PWMs get defined for individual TF family members when, in reality, they bind to the same DNA motif, and different PWMs even get defined for the same factor. As typified by a few representative members in Figure 2D, the entire ETS family could be condensed down to the point where all ETS factors recognized the same motif, except for PU.1, which has a distinctive motive. Using this approach, we compiled a list of 80 PWMs that accounted for all the binding sites for the 284 TF genes that were expressed in any of the AML samples or in PBSCs included in this study (Supplementary Table 1). This table can now be used by others constructing similar networks.

**Figure 3.**
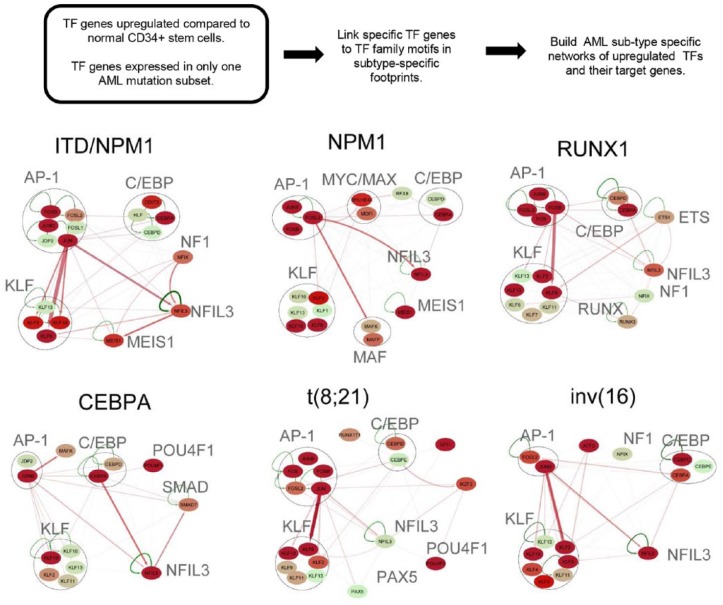
Identification of transcription factor networks driving AML-type-specific gene expression. AML-specific transcription factor (TF) networks are derived by identifying TF genes that are upregulated in AML and then linking these to their motifs where they are footprinted in an AML-specific manner in other genes. Members of deregulated transcription factor families binding to the same motif are depicted as nodes contained within a circle. Each node includes at least 1 member that is upregulated, relative to normal cells or other types of AML, plus all other members of the same family that are expressed. Arrows going outwards from the node highlight footprinted motifs in other individual genes generated by any member of this TF family. Where possible, the footprinted DHS was linked to the target gene using promoter capture HiC data. The relative expression level (FKPM) for the individual genes is depicted in color for low (green), moderate (orange), and high (red) levels of mRNA expression. AML indicates acute myeloid leukemia; DHS, DNase I hypersensitive site.

In steps C and D of Figure 1, RNA-Seq and promoter capture HiC data were used to infer functional connections between TF footprints in DHSs and the active genes they control. In the absence of HiC data, other GRN studies are obliged to simply associate DHSs with the nearest genes. In our hands, only 50% of promoter-DHS interactions were in fact with the nearest gene. We began by showing that 85% of distal DHSs exhibited interactions with at least 1 promoter. For the other 15%, we fell back to pairing DHSs with the nearest gene. For those DHSs that were deregulated in an AML-specific manner, we did find that ~70% of them interacted with the nearest promoter. Finally, in step E of Figure 1, we combined footprinting, TF gene expression, and, where possible, promoter capture HiC data to construct TF networks in different AML subtypes by linking occupied binding motifs within TF genes to the specific TF families recognizing these motifs. Figure 3 summarizes a subset of the most significant interactions defined by this approach. These networks were further validated using siRNA-mediated knock-down of the expression of critical TF genes in AML cells. Most significantly, the AP-1 TF family formed a prominent regulatory node in all the AML subsets, and the expression of a dominant negative form of AP-1 was sufficient to block both FLT3-ITD and t(8;21) AML tumor formation in mice.^12^

To add an extra level of complexity to the network analyses, we also determined which different TFs frequently cooperate with each other in AML-type-specific patterns. We therefore searched for significant co-localizing occupied motifs within 50-bp windows. This analysis showed that motif occupancy patterns are highly AML type-specific. Different AML-subtype-specific TF networks were identified, highlighting a number of TF genes that form regulatory nodes in AML-subtype-specific TF networks of upregulated TF genes that change expression at least threefold compared with normal cells and appear to have AML-specific pathways and roles.

In conclusion, this study integrated multiple types of data to identify the major AML subtype-specific TF networks. It showed that a specific subset of leukemic drivers is primarily responsible for controlling the regulatory phenotypes by creating specific TF regulatory and signaling networks that are different from those in normal cells. It highlighted a number of aberrantly upregulated TF genes, such as *FOXC1* and *POU4F1*, which appear to have AML subtype-specific roles, some of which were validated as potential therapeutic targets. Moreover, our data contain many downstream effector genes regulated by these TFs, which represent a rich resource of potential drug targets. We believe that, based on our study, personalized medicine in AML has now become a step closer.

## Supplemental Material

Supplementary_Table_S1_ – Supplemental material for Rewiring of the Transcription Factor Network in Acute Myeloid Leukemia Click here for additional data file. Supplemental material, Supplementary_Table_S1_ for Rewiring of the Transcription Factor Network in Acute Myeloid Leukemia by Salam A Assi, Constanze Bonifer and Peter N Cockerill in Cancer Informatics
